# Plant stress physiology: safeguarding food security in a changing climate

**DOI:** 10.1093/jxb/eraf340

**Published:** 2025-11-04

**Authors:** Adriano Nunes-Nesi, Wagner L Araújo, Agustin Zsögön

**Affiliations:** National Institute of Science and Technology on Plant Physiology Under Stress Conditions, Departamento de Biologia Vegetal, Universidade Federal de Viçosa, 36570-900,Viçosa, Minas Gerais, Brazil; National Institute of Science and Technology on Plant Physiology Under Stress Conditions, Departamento de Biologia Vegetal, Universidade Federal de Viçosa, 36570-900,Viçosa, Minas Gerais, Brazil; National Institute of Science and Technology on Plant Physiology Under Stress Conditions, Departamento de Biologia Vegetal, Universidade Federal de Viçosa, 36570-900,Viçosa, Minas Gerais, Brazil

**Keywords:** Abiotic stress, biotic stress, crop resilience, ecophysiology, yield

## Abstract

The brunt of climate change in the coming decades will be felt most acutely in the Global South, comprising Asia, Africa, and Latin America, where nearly two-thirds of the world’s crop yield by volume is produced annually (Zsögön *et al*., 2022). Brazil, for instance, is the world’s largest producer of soybeans, sugarcane, coffee, and orange juice (FAO, 2022). Training the next generation of crop physiologists is therefore essential to address these pressing challenges. At the Graduate Program in Plant Physiology at the Federal University of Viçosa, in Minas Gerais, Brazil, we are strongly committed to this mission. In line with this commitment, from 7 to 11 October 2024 we hosted the 19th Brazilian Congress of Plant Physiology (https://cbfv.sbfv.org.br/). This longstanding event brings together domestic and international students, researchers, and stakeholders from both academia and industry. This Special Issue features a diverse set of contributions, some of which were presented during the event, which was generously sponsored by the Journal of Experimental Botany (JXB).

Following a brief welcome and introduction to the event by the Head of the Organizing Committee, Adriano Nunes-Nesi (Federal University of Viçosa), and the President of the Brazilian Society for Plant Physiology, Danilo Daloso (Federal University of Ceará, Brazil), the meeting was kicked off by a plenary talk by John Lunn (Max Planck Institute for Molecular Plant Physiology, Germany), who provided a highly engaging outline of the global challenges for plant biology in the 21^st^ century. The next day the plenary lectures of the Photosynthesis and Metabolism session were presented by Andreas Weber (Heinrich Heine University Düsseldorf, Germany), Elizabete Carmo-Silva (Lancaster University, UK), Stefan Timm (University of Rostock, Germany), and Jaume Flexas (University of Balearic Islands, Spain). This session provided a comprehensive view of how understanding and manipulating photosynthetic processes and stress responses can contribute to improving crop productivity and resilience in the face of climate change.

The afternoon session of day two, on Plant Growth and Development, included plenary talks by Angus Murphy (University of Maryland, USA), Magdalena Rossi (University of São Paulo, Brazil), and Maria Laura Vidoz (Botanical Institute of the Northeast, Argentina), which collectively illustrated how molecular transport, transcriptional networks, and stress physiology are tightly interconnected. Letícia dos Anjos, representing the Brazilian Network of Women in Plant Sciences (BNWPS), presented an overview of the past, present, and future of gender equality in Brazilian plant sciences. Her talk highlighted both the progress achieved and the persistent challenges faced by women in the field of plant sciences, especially in Brazil.

The next day started with the Plant Hydraulics and Water Relations session, which explored how plant hydraulic function underpins responses to environmental stress, with insights ranging from the cellular level to whole ecosystems, and spanning biomes from deserts to tropical forests. The plenary talks were held by Christine Scoffoni (California State University Los Angeles, USA), Sandra Bucci (National University of Patagonia, Argentina), Augusto Franco (University of Brasília, Brazil), and Rafael Oliveira (State University of Campinas, Brazil). In the afternoon, the Plant–Pathogen Interactions session was run in parallel with ‘Physio-Pop’, a space designed to promote science outreach by fostering interaction between students from public schools in the Viçosa region and conference participants. The Plant–Pathogen Interactions session was opened by Jürgen Zeier (Heinrich Heine University Düsseldorf, Germany), followed by Marcelo Lattarulo Campos (Federal University of Mato Grosso, Brazil), and Elizabeth Fontes (Federal University of Viçosa, Brazil), and closed by Leonardo de la Fuente (Auburn University, USA), who highlighted the intricate balance plants maintain between immunity, metabolism, and growth, as well as the evolutionary arms race between plants and their pathogens or pests.

Day four of the meeting began with a morning session on Genetics and Molecular Plant Physiology, and plenary lectures by José Jiménez-Gómez (Polytechnic University of Madrid, Spain), Luciano Freschi (University of São Paulo, Brazil), Raquel Chan (UNL-CONICET Joint Research Institutes, Argentina), and Yunde Zhao (University of California San Diego, USA). Together, these lectures complemented each other smoothly to demonstrate how the study of natural genetic variation, molecular signaling, gene regulation, and precise genome editing can provide tools to develop crops that are more resilient, productive, and suitable for future agricultural challenges. The afternoon session on Stress Physiology and Systems Biology explored how metabolic regulation can improve photosynthetic efficiency in the face of climate change. The plenaries were held by Carlos Figueroa (Instituto de Agrobiotecnología del Litoral, Argentina), Fabiana Drincovich (Universidad Nacional de Rosario, Argentina), and Xurxo Gago (University of Balearic Islands, Spain). Every session concluded with thoughtful and engaging questions from the audience, mainly undergraduate and graduate students.

The final day of the conference was highlighted by a round table on the topic of Equity in open access publications: Opportunities and Barriers for Brazilian Plant Science, and included a highly engaging Q&A session with John Lunn (Editor-in-Chief of the *Journal of Experimental Botany*), Yunde Zhao (Editor-in-Chief of *Plant Physiology*), Susanne Brink (Editor-in-Chief of *Trends in Plant Science*), Raquel Chan (Editor-in-Chief of *Plant Science*), and Gustavo Habermann (Editor-in-Chief of *Theoretical and Experimental Plant Physiology*). The final plenary talk was given by Fábio DaMatta (Federal University of Viçosa), who recounted the history of the Graduate Program in Plant Physiology at the Federal University of Viçosa, from its inception by Moacyr Maestri, and presented the award named in his memory to the selected speakers of the Young Researcher Session: Francisco Bruno Silva Freire, Valeria Lima, and Kleiton Lima de Godoy Machado.

Almost 700 people attended the Congress, among which 180 were professionals, 142 graduate students, and 192 undergraduates. The talks were given by 27 speakers from seven different countries. Over 476 posters were presented over days two and three of the event. Although there was no formal award for best poster, special recognition was given to Erica Lopes Batista (Colégio Coluni, Brazil), a local high school student who conducted research using tomato mutants and delighted the attendees with the confident and clear delivery of her results. In summary, the event was an unmitigated success with record attendance and a vibrant exchange of ideas among students, researchers, and industry professionals from Brazil and abroad.

Based on the success of this congress, 18 Research papers, 11 Reviews, and one Viewpoint are compiled in this collection to reflect the dynamic and multidisciplinary nature of current plant stress physiology. The articles are organized broadly in three themes that encompass the scope of the research presented during the event. These are: (i) Abiotic stress tolerance and climate adaptation; (ii) Water use, stomatal biology, and photosynthesis; and (3) Genetic and molecular mechanisms of stress and immunity.

## Abiotic stress tolerance and climate adaptation

In response to the urgent need for improving crop resilience in a changing climate, several contributions in this Special Issue explore physiological and genetic mechanisms underpinning tolerance to abiotic stresses such as heat (Raineri *et al*., 2025), high irradiance (Dieckmann *et al*., 2025), flooding and drought (Singh *et al*., 2025). These studies include integrative multi-omic analyses in sorghum (Watson-Lazowski *et al*., 2025), functional genomics in barley (Chakraborty *et al*., 2025) and rice (Zhu *et al*., 2025), and the exploration of wild relatives to enhance heat resilience in quinoa (Xu *et al*., 2025). Additionally, the potential trade-offs between growth and stress tolerance that may have emerged during domestication are critically examined across *Poaceae* species (Yun *et al*., 2025). Recent advances in high-throughput phenotyping using UAVs (unmanned aerial vehicles) combined with machine learning have also improved our ability to predict root traits critical for stress tolerance in barley under field conditions (Alahmad *et al*., 2025).

Complementing the findings above, a series of reviews provide insights into the molecular mechanisms of abiotic stress tolerance. The roles of mitochondrial carrier proteins in plant responses to abiotic stress further elucidate intracellular adaptations (Monteiro-Batista *et al*., 2025). Advances in understanding metal homeostasis, such as the crosstalk of iron with zinc, copper, phosphorus, and nitrogen, underscore the complexity of nutrient regulation under stress conditions (Wairich *et al*., 2025). Moreover, oxalate oxidase enzymes have been implicated in both stress tolerance and industrial applications, expanding the functional repertoire of stress-associated proteins (Gómez-Espinoza *et al*., 2025). Studies of plant resilience to abiotic stress reveal the beneficial role of silicon in mitigating drought and metal(loid) toxicity (Mora-Sanhueza *et al*., 2025), while plant primary metabolism adjustments highlight critical shifts in response to climate change (Bulut *et al*., 2025). Lastly, an updated perspective by Ying (2025) on chloroplast plastoglobule-associated proteins sheds light on their pivotal roles in plant responses to abiotic stress, revealing new molecular targets for crop improvement.

## Water use, stomatal biology, and photosynthesis

A central theme of the Congress was understanding how plants balance water use and carbon assimilation under environmental stress conditions. Contributions in this section examined anatomical and functional aspects of stomatal traits (Zhen *et al*., 2025) and hormonal regulation of stomatal behavior (Huang *et al*., 2025). This section also features innovative insights into photosynthetic efficiency in maize (Yang *et al*., 2025) and the unique cellular organization of *Moricandia arvensis*, a C_3_–C_4_ intermediate species (Triesch *et al*., 2025). López-González *et al*. (2025) highlight how species-specific phyllosphere responses to external pH changes contribute to molecular and ecological stress responses at the leaf surface interface, an emerging frontier in plant immunity and microbiome interactions. Further highlighting photosynthesis under stress, a review article by Clemente-Moreno *et al*. (2025) discusses the interplay between cell wall dynamics and photosynthetic performance to elucidate how structural adjustments support tolerance mechanisms. Photoperiod insensitivity in crops is reviewed by González-Delgado *et al.* (2025), revealing implications for water use and photosynthetic adaptation in varying environments. Lastly, insights from plants thriving in extreme habitats shed light on stomatal regulation and photosynthetic adaptations essential for survival under water-limited conditions (Flexas *et al*., 2025).

## Genetic and molecular mechanisms of stress and immunity

Advances in molecular plant biology are exemplified by studies uncovering signaling networks involved in nutrient homeostasis (Roychowdhury *et al*., 2025), biotic stress responses, and plant immunity (Nerva *et al*., 2025). Key findings include the role of antifungal cytokines in immune signaling (Lintz *et al*., 2025) and jasmonate-mediated defense pathways in rice (Wang *et al*., 2025 ), highlighting how molecular tools and genetic resources are being harnessed to tackle both biotic and abiotic challenges. Yu *et al*. (2025) review how histone methylation has revealed epigenetic regulation as a crucial layer of plant responses to abiotic stresses. The functions of trihelix transcription factors further illustrate the genetic control of development and stress resilience (Ibarra and Reynoso, 2025). Additionally, Pinheiro *et al*. (2025) review how non-host resistance during plant–insect interactions provide novel insights into immunity and defense strategies beyond classical pathogen responses.

Together, these contributions demonstrate the collaborative spirit that characterizes the Brazilian plant physiology community and its international partners. We extend our gratitude to everyone who attended the event and to all our sponsors for their generous support. We hope this Special Issue not only showcases the growing scientific contributions from the Global South but also inspires the next generation of crop physiologists committed to addressing the pressing the pressing agricultural challenges of our time.
